# Supplementary material for: Chemical Modification of Auranofin Yields a New Family of Anticancer Drug Candidates: The Gold(I) Phosphite Analogues
Source: Molecules. 2023 Jan 20;28(3):1050. doi: 10.3390/molecules28031050 (PMC9920260; doi:10.3390/molecules28031050)
Supplement: Supplementary file 1 [file molecules-28-01050-s001.zip › molecules-2160445-supplementary.pdf]

Chemical modification of auranofin yields a new family of anticancer drug candidates: the gold(I) Phosphite analogues

Damiano Cirri, Andrea Geri, Lara Massai, Michele Mannelli, Tania Gamberi, Chiara Gabbiani, Francesca Magherini, Matteo Becatti, Alessandro Pratesi and Luigi Messori

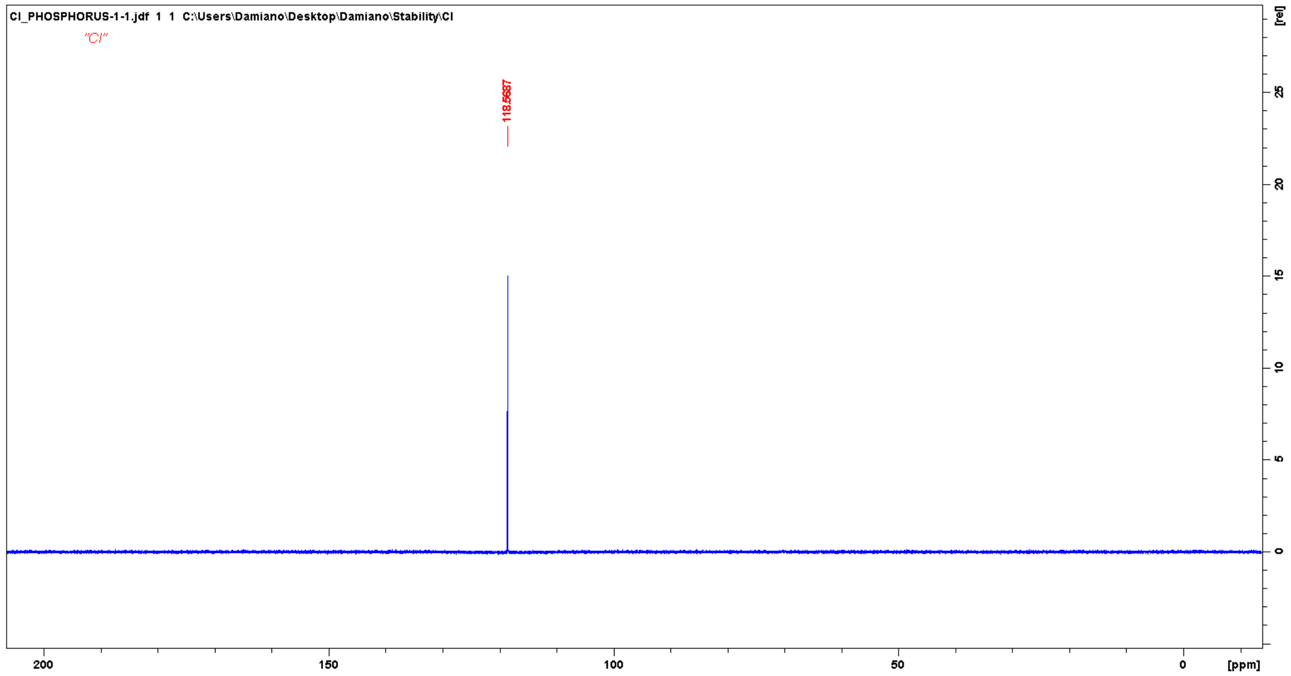

Figure S1: ^{31}PNMR spectrum performed at t_0 on $AuP(OCH_3)_3Cl$. Solvent: MeOD-d_4/H_2O 1:1

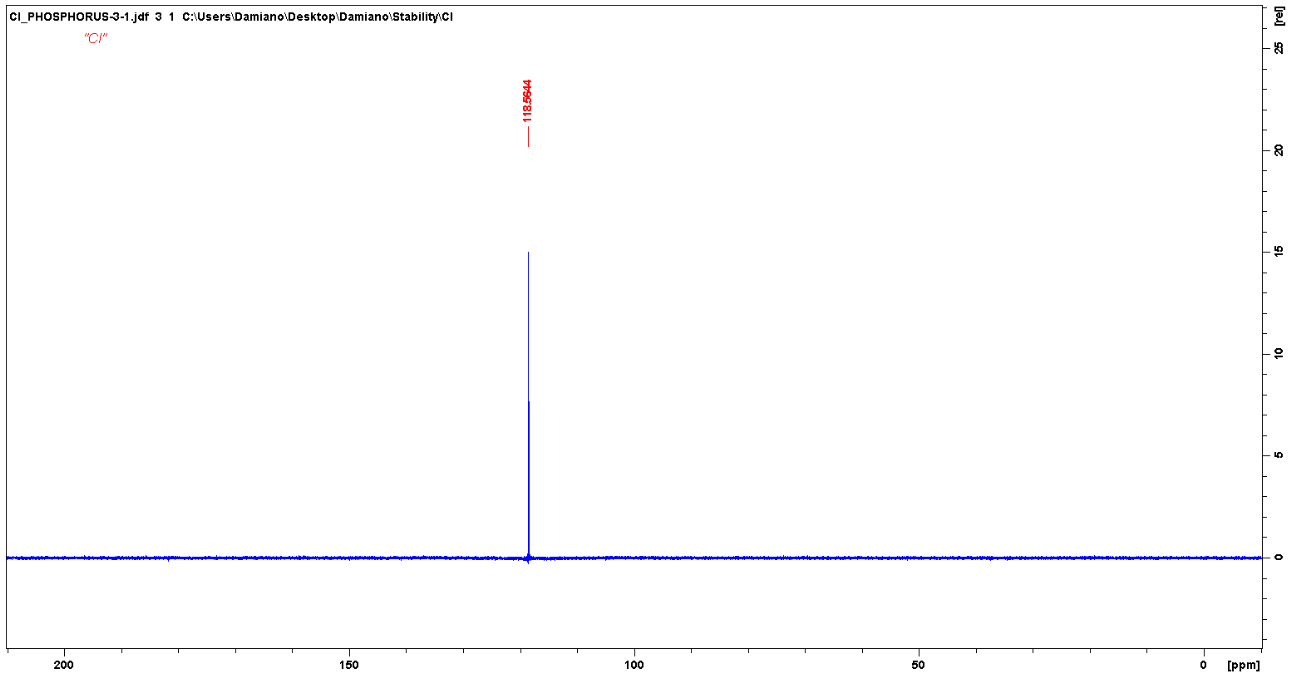

Figure S2: ^{31}PNMR spectrum performed at 72h on $AuP(OCH_3)_3Cl$. Solvent: MeOD-d_4/H_2O 1:1

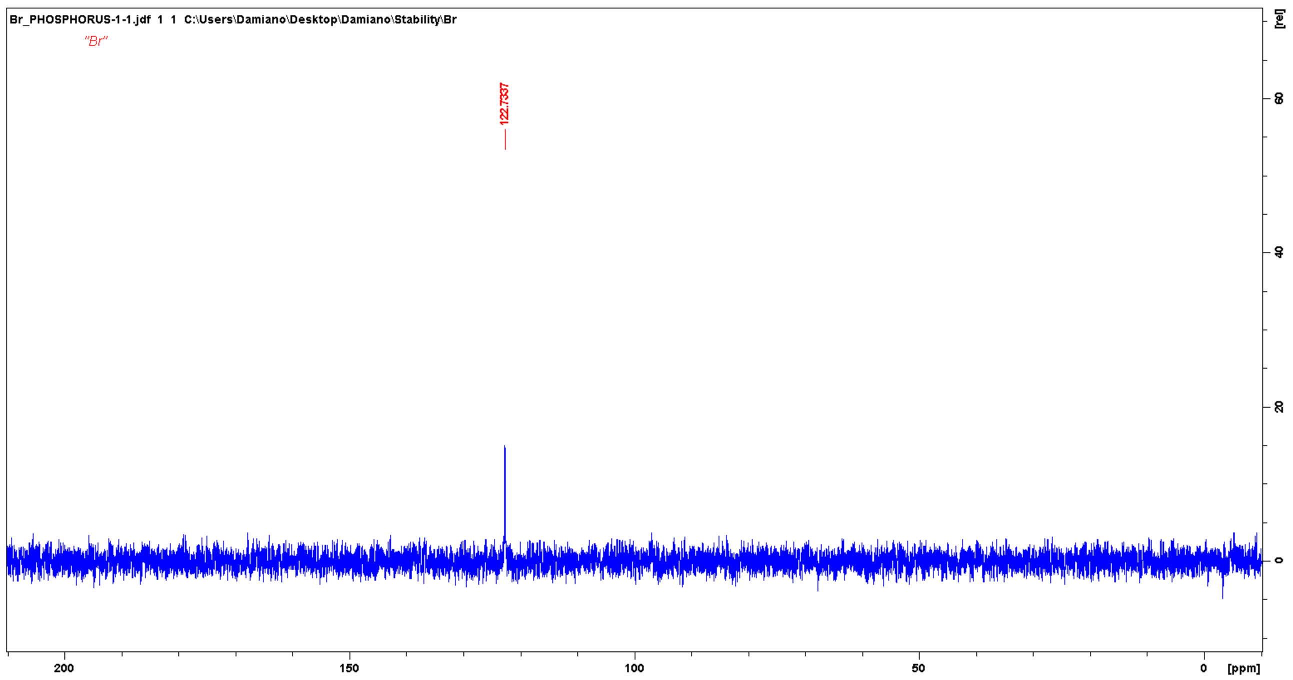

Figure S3: ^{31}PNMR spectrum performed at t_0 on $AuP(OCH_3)_3Br$. Solvent: MeOD-d_4/H_2O 1:1

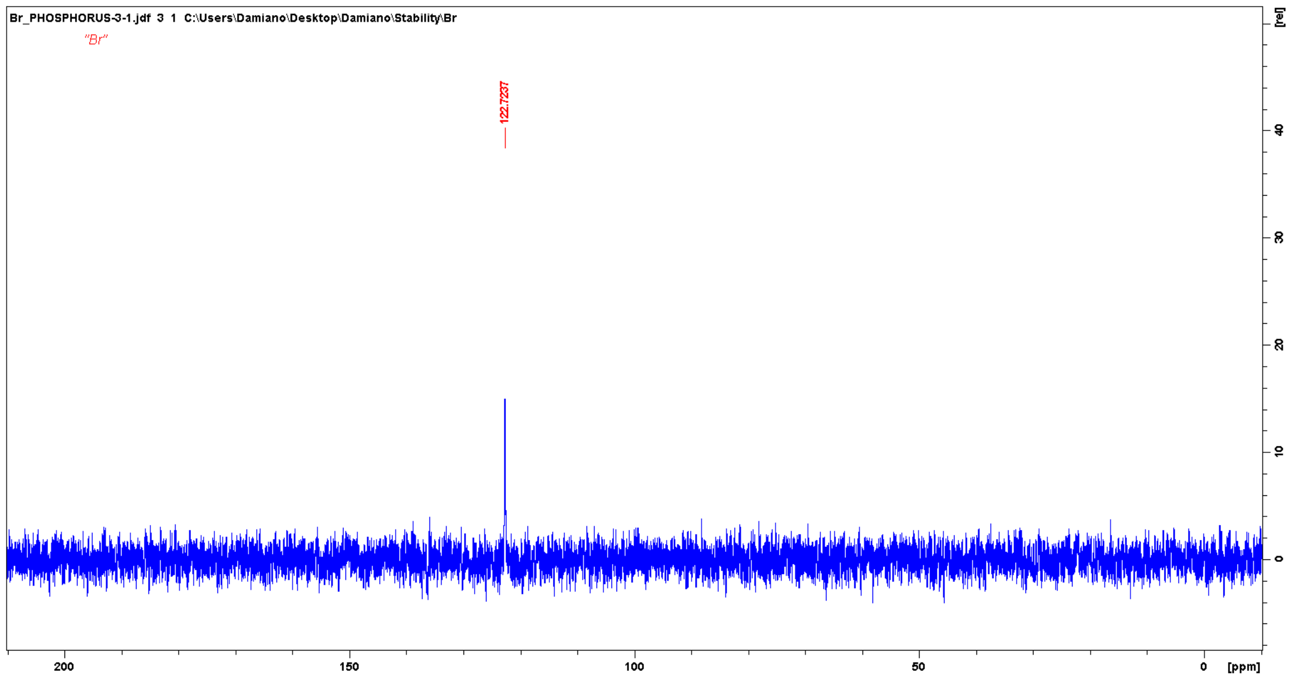

Figure S4: ^{31}PNMR spectrum performed at 72h on $AuP(OCH_3)_3Br$. Solvent: MeOD-d_4/H_2O 1:1

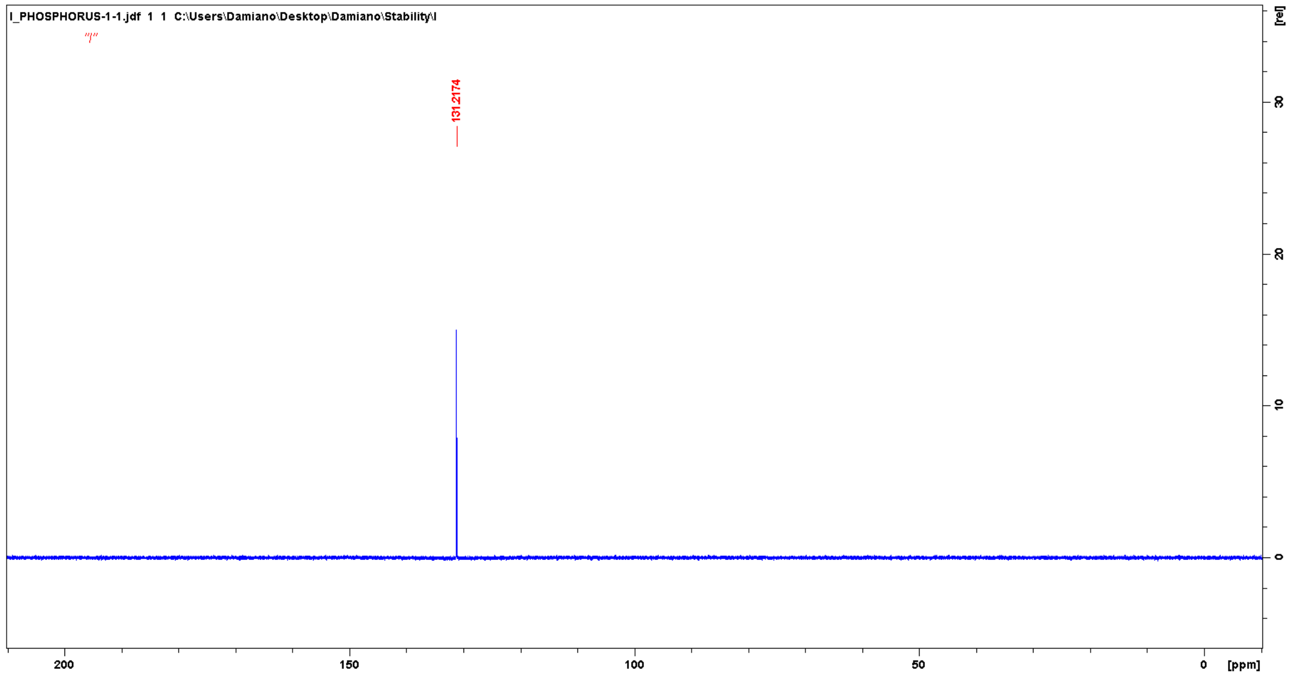

Figure S5: ^{31}PNMR spectrum performed at t_0 on $AuP(OCH_3)_3I$. Solvent: MeOD-d_4/H_2O 1:1

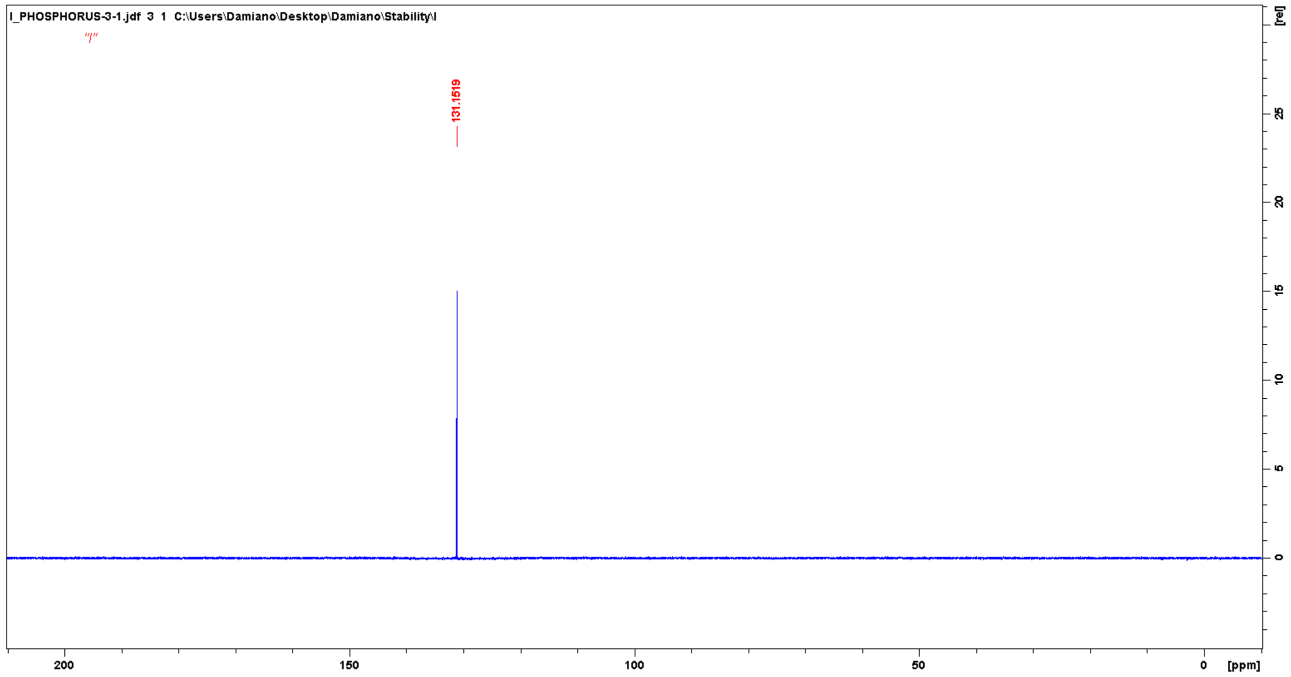

Figure S6: ^{31}PNMR spectrum performed at 72h on $AuP(OCH_3)_3I$. Solvent: MeOD-d_4/H_2O 1:1

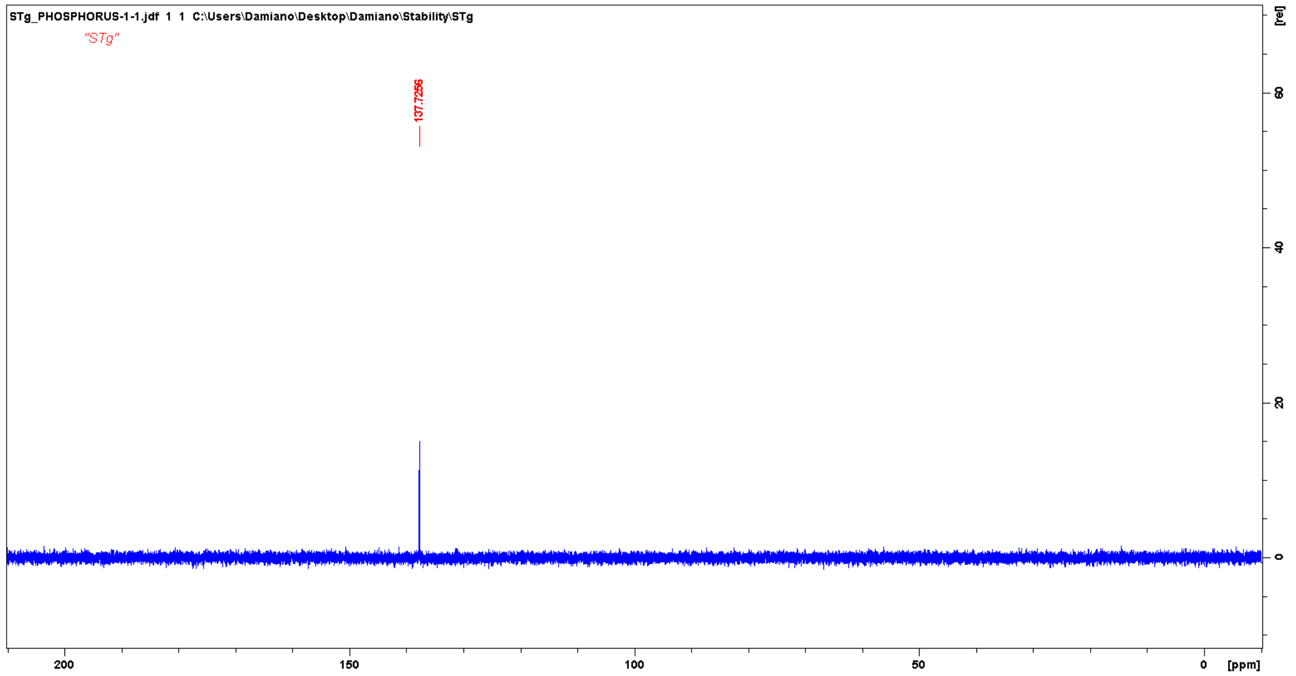

Figure S7: ^{31}PNMR spectrum performed at t_0 on AuP(OCH_3)$_3$STga. Solvent: MeOD-d_4/ H_2O 1:1

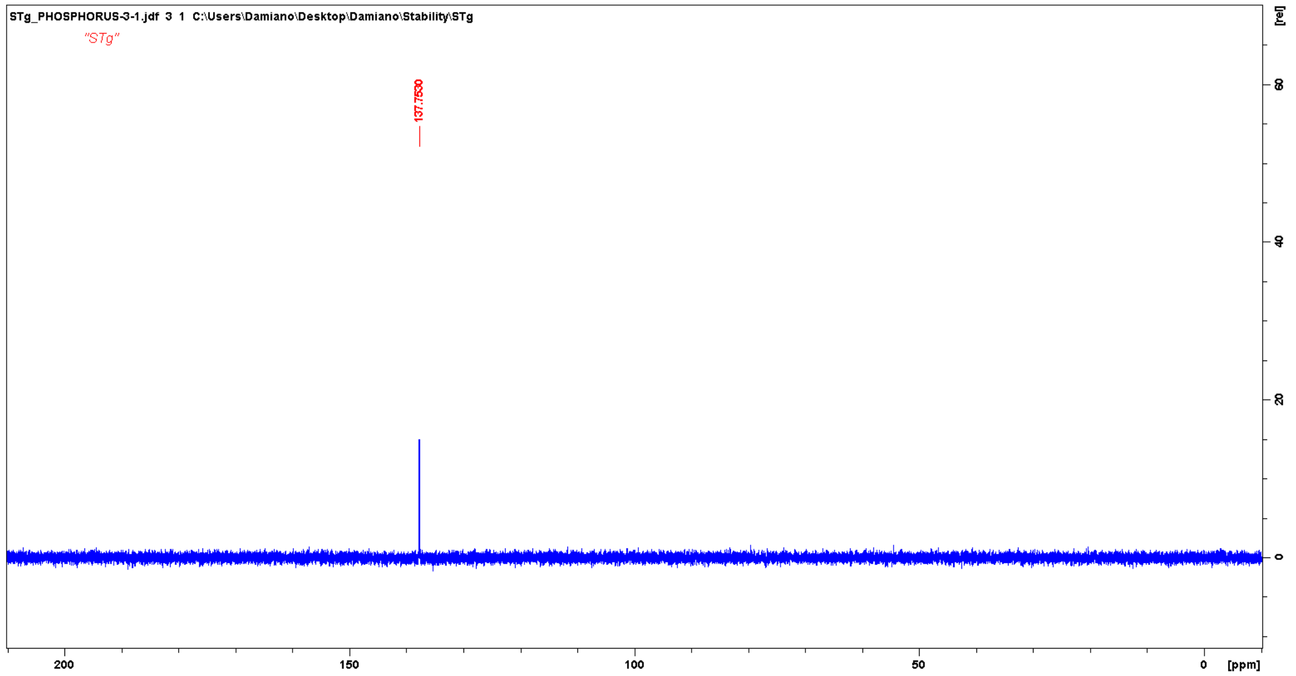

Figure S8: ^{31}PNMR spectrum performed at 72h on AuP(OCH_3)$_3$STga. Solvent: MeOD-d_4/ H_2O 1:1

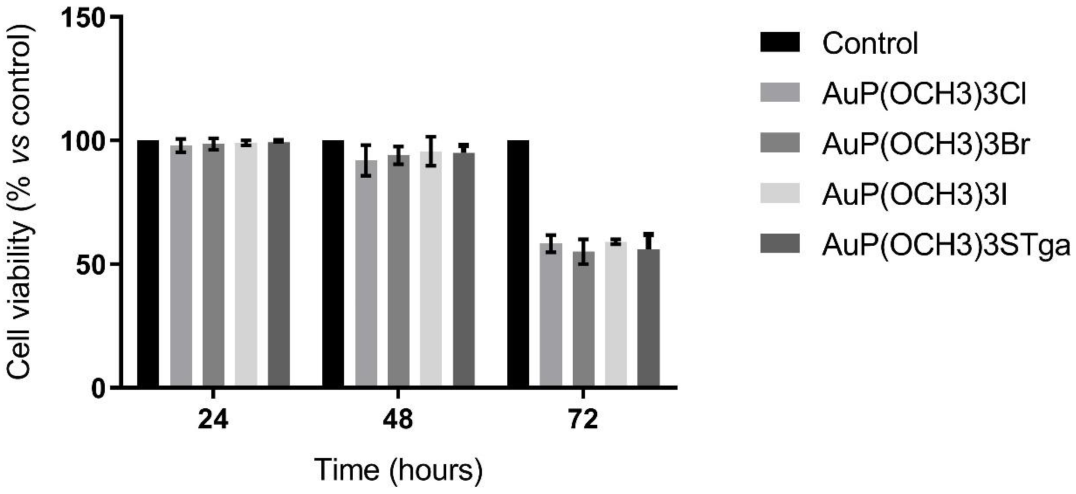

Figure S9: Cell Viability Assay. MTT time course assay at 24, 48, and 72 h of drug exposure by using their 72-h exposure IC50 doses.
